# Supplementary material for: The Antimicrobial Effects of Bacterial Cellulose Produced by Komagataeibacter intermedius in Promoting Wound Healing in Diabetic Mice
Source: Int J Mol Sci. 2022 May 13;23(10):5456. doi: 10.3390/ijms23105456 (PMC9142012; doi:10.3390/ijms23105456)
Supplement: Supplementary file 1 [file ijms-23-05456-s001.zip › ijms-1700926-supplementary.pdf]

Table S1. The Abbreviations of Skin Surface characteristics in this manuscript.

Abbreviations	Definition
CIE-a*	Commission Internationale d'Eclairage a*: the value of green-red color on the detected wounds.
CIE-b*	Commission Internationale d'Eclairage b*: the value of blue-yellow color on the detected wounds.
CIE-L*	Commission Internationale d'Eclairage L*: the value of white color on the detected wounds.
RH	Relative humidity: The relative humidity of the wounds
TEWL	Transdermal water loss: The amount of water that passively evaporates through skin to the external environment
Wound R.	Wound recovery: The recovery rate of the wounds
